# A Role for Glycine Transporter‐2 Inhibitors in Regulating Accumbal Dopamine Levels and Voluntary Alcohol Intake in Rats

**DOI:** 10.1111/acer.70418

**Published:** 2026-09-21

**Authors:** Yasmin Olsson, Klara Danielsson, Ana Domi, Helga Lidö, Linda Engström Ruud, Mia Ericson, Bo Söderpalm

**Affiliations:** ^1^ Beroendekliniken, Sahlgrenska University Hospital Gothenburg Sweden; ^2^ Addiction Biology Unit, Department of Psychiatry and Neurochemistry, Institute of Neuroscience and Physiology, Sahlgrenska Academy University of Gothenburg Gothenburg Sweden; ^3^ Department of Neurology Sahlgrenska University Hospital Gothenburg Sweden; ^4^ Department of Physiology, Institute of Neuroscience and Physiology, Sahlgrenska Academy University of Gothenburg Gothenburg Sweden

**Keywords:** alcohol addiction, dopamine, ethanol consumption, glycine transporter, nucleus Accumbens

## Abstract

**Background:**

Glycinergic neurotransmission has been suggested as a tentative pharmacological treatment target for Alcohol Use Disorder (AUD), a devastating brain disease with limited treatment options that involves reduced dopamine (DA) activity in the nucleus Accumbens (nAc) and loss of control of alcohol intake. Glycine receptors (GlyRs) in the nAc, a pivotal part of the brain reward circuitry, are involved in regulating basal and alcohol‐induced accumbal DA levels as well as voluntary alcohol intake in rats.

**Methods:**

This study in rats examined if inhibition of the glycine transporter 2 (GlyT‐2, SLC6A5), present within glycinergic synapses, alters (1) accumbal DA and glycine content as measured by in vivo microdialysis coupled to HPLC and (2) the alcohol deprivation effect (ADE) in a two‐bottle‐choice alcohol consumption paradigm. Finally, (3) the presence of GlyT2‐expressing fibers and GlyRs in the nAc was examined by immunohistochemistry (IHC).

**Results:**

In brief, the two partial GlyT2‐inhibitors N‐Arachidonyl‐Glycine (NAGly, 10 mg/kg i.p.) and Opiranserin (20 mg/kg i.p.), but not the full GlyT2‐inhibitor Org 25543, slightly elevated accumbal DA levels, while no effects on extrasynaptic glycine levels were observed. The reversible GlyT1‐inhibitor Bitopertin (10 mg/kg p.o.) produced a pronounced glycine and DA elevation but combined GlyT1‐ and GlyT2 inhibition did not yield any statistically significant additive effects. In the alcohol consumption study, NAGly abolished the ADE as compared to placebo treatment. Finally, histology confirmed the presence of GlyT2 and GlyR in the nAc, although with a higher signal for GlyT2 in, for example, septal and basal forebrain regions.

**Conclusions:**

Overall, this study indicates that synaptic GlyRs are involved in regulating nAc DA levels and alcohol intake in rats, but probably to a lesser extent than extrasynaptic GlyRs. Further studies that specifically target GlyT2 in, for example, the nAc and septal regions and that utilize refined techniques for measuring synaptic glycine levels are warranted to better characterize the role of glycinergic innervation in the reward circuitry and for regulating alcohol intake.

## Graphical abstract

Partial inhibition of glycine transporter‐2 (GlyT2) elevates basal dopamine levels in the nucleus accumbens and abolishes the alcohol deprivation effect in rats. The findings reveal a role for GlyT2‐expressing glycinergic neurons in regulating mesolimbic dopamine signaling and relapse‐like alcohol drinking, highlighting GlyT2 as a potential target for Alcohol Use Disorder treatment.

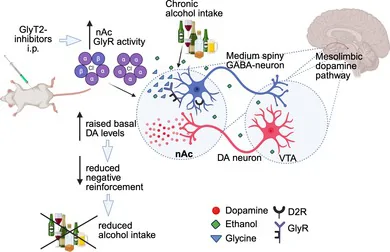

## 1 Introduction

Alcohol Use Disorder (AUD) is a relapsing brain disorder characterized by difficulties in regulating alcohol intake despite negative consequences and development of a dysphoric emotional state when hindered to consume alcohol (Hasin et al. 2013; Koob and Volkow 2016). Worldwide, the lifetime prevalence of AUD is estimated to be 8.6% (Glantz et al. 2020), constituting one of the most widely prevalent psychiatric disorders (Rehm and Shield 2019). Alcohol use in itself significantly contributes to the global burden of disease as it is attributed approximately 5% of all disability‐adjusted life years (DALYs) and premature deaths (Shield et al. 2020). Furthermore, harm inflicted by alcohol use is dose‐dependently related to the amount consumed (Rehm et al. 2017), and individuals that suffer from AUD present the highest levels of drinking. Consequently, life expectancy is reduced by approximately 22 years in individuals with AUD with very high drinking risk levels (according to WHO criteria; van den Brink et al. 2018). Approved pharmacotherapies for AUD include disulfiram, a form of aversion therapy; acamprosate and naltrexone, the latter compounds being hypothesized to interfere with brain reward mechanisms and thereby aiding in the maintenance of abstinence from alcohol and/or in reducing its consumption (Kranzler and Hartwell 2023). However, effect sizes for AUD medications are small (Jonas et al. 2014; Maisel et al. 2013), but may be improved if identifying new pharmacological targets that are specifically implicated in the reinforcing properties of alcohol.

Acute alcohol consumption raises dopamine (DA) levels in the nucleus Accumbens (nAc) in the brain reward circuitry, an event hypothesized to be a neurobiological correlate to its positive reinforcing properties (di Chiara and Imperato 1988). In contrast, results from studies in rats (Feltmann et al. 2016; Weiss et al. 1996) and in humans (Heinz et al. 2004; Volkow et al. 2007) suggest that chronic alcohol intake involves perturbations in DA transmission in the nAc (ventral striatum in humans), yielding a hypothesized hypodopaminergic state. The mitigated function of the mesolimbic dopamine system is presumably involved in the negative emotional state in addicted individuals, which perpetuates AUD through negative reinforcement (Koob and Volkow 2016).

Our research group has previously presented evidence suggesting that the inhibitory, ligand‐gated glycine receptor (GlyR) present on, for example, spiny projection neurons in the nAc, is pivotal for regulating basal and alcohol‐induced DA release in the nAc, presumably via a mechanism that involves disinhibition of DA neurons projecting from the ventral tegmental area (VTA) to the nAc (Söderpalm et al. 2017). Indeed, local perfusion (into the nAc) and systemic treatment with the endogenous GlyR agonist glycine or with indirect GlyR agonists (i.e., glycine transporter‐1 (GlyT1)‐inhibitors), raise basal and attenuate alcohol‐induced DA release in the rat nAc, while these effects are abolished following local perfusion with the GlyR‐antagonist strychnine (Olsson et al. 2021; Söderpalm et al. 2017). Furthermore, alcohol intake in rats is reduced following local and systemic treatment with GlyR agonists (Olsson et al. 2021; Söderpalm et al. 2017; Vengeliene et al. 2018), probably due to alterations in accumbal DA output and ensuing effects on positive and negative reinforcement to consume alcohol.

GlyRs are expressed in diverse regions within the CNS, including the spinal cord, brain stem, hippocampus and striatum and can, depending on brain region and subunit constellation, both be intra‐ or extrasynaptically located (Burgos et al. 2016). Glycine, and to a lesser extent several other amino acids, activate the GlyR, whereas alcohol acts as a positive allosteric modulator on certain GlyR subtypes (Burgos et al. 2016). Homeostatic intra‐ and extrasynaptic concentrations of glycine in the CNS are maintained by the GlyT1, the glycine transporter 2 (GlyT2) and the alanine‐serine‐cysteine‐1 transporter (Asc‐1) (Eulenburg and Hülsmann 2022). GlyT1s are widespread within the CNS, including the nAc, and when present on presynaptic neurons or, as more frequent, on adjacent glial cells, they primarily remove glycine from extracellular compartments (Eulenburg et al. 2005). Thereby, the GlyT1 constitutes a pharmacological treatment target in conditions in which enhanced glycinergic transmission is desired, for example, alcohol addiction and pain (Cioffi 2021; Harvey and Yee 2013). Indeed, GlyT1‐inhibition in rat models has been shown to raise extracellular glycine and DA levels in the nAc and to reduce alcohol intake. However, it failed to prevent alcohol relapse to any larger extent than placebo in individuals with AUD (de Bejczy et al. 2014).

**FIGURE 1:**
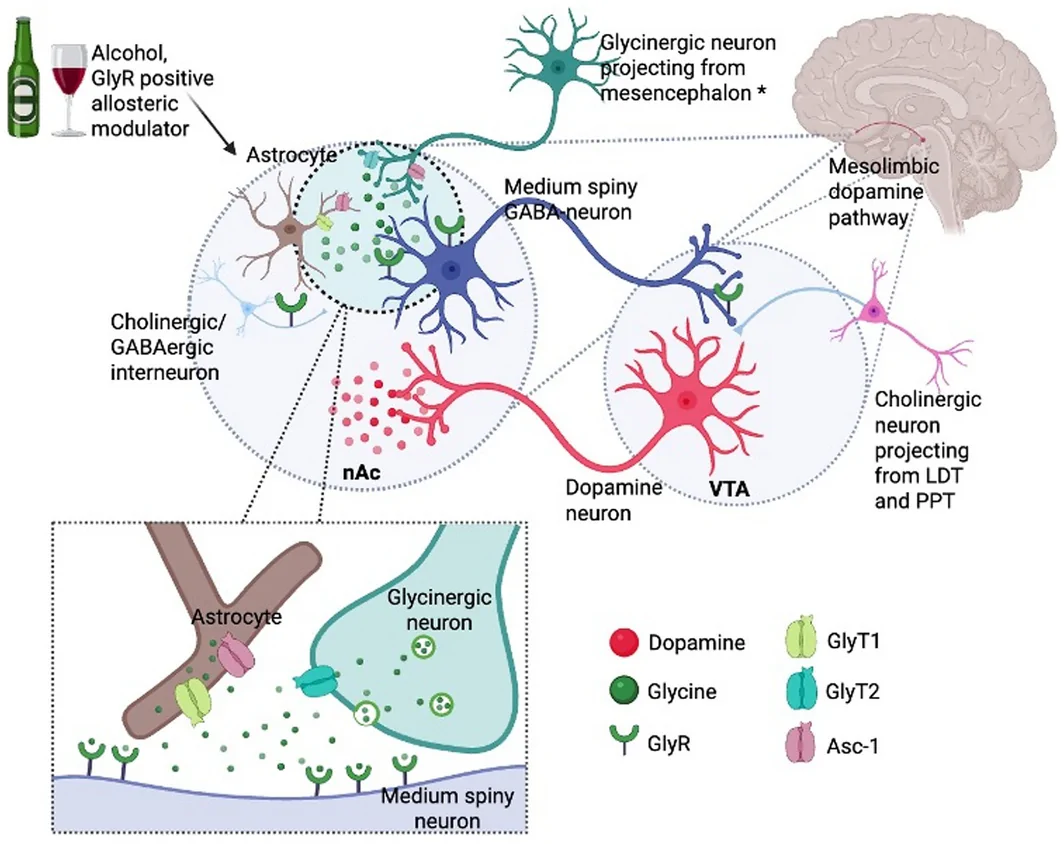
Schematic overview of hypothesized glycinergic signaling in the nAc. GlyRs are present on GABA‐ergic medium spiny neurons (MSNs), cholinergic and/or GABA‐ergic interneurons in the nAc and on end‐terminals of MSNs or GABA‐ergic interneurons in the ventral tegmental area (VTA). Accumbal MSNs exhibit both synaptic and non‐synaptic GlyRs. Glycine is the primary GlyR agonist and alcohol acts as a positive allosteric modulator on certain GlyR subunit compositions. The transporter proteins GlyT1, GlyT2, and Asc‐1 regulate extracellular glycine levels. GlyT1 is, within the nAc, present on astrocytes, where it primarily transports glycine from the extracellular space into the astrocyte, although it may also operate in a reversed mode. Asc‐1 is present on both glial cells and at the presynapse and transports glycine from glial cells to the extracellular space. GlyT2 is exclusively expressed at the end‐terminal of glycinergic neurons, serving to transport glycine back into the terminals thereby enabling synaptic vesicles to be refilled. Glycine in the nAc derives from either synaptic release by glycinergic projection neurons or from reversed transport by glial GlyT1s. In addition, de novo synthesis or conversion/degradation of glycine in glial mitochondria and import and export of glycine across the blood–brain barrier also impact extracellular glycine concentrations. Activation of GlyRs (in the nAc) hyperpolarizes MSNs projecting to the VTA, whereby its tonic inhibition of DA‐ergic neurons projecting back to the nAc is reduced, which ultimately raises DA levels in the nAc. Pharmacological interventions that enhance GlyR signaling raise basal DA levels and are hypothesized to transiently desensitize the GlyRs, thereby preventing alcohol from producing any further elevation in accumbal DA levels. It is proposed that raised DA output that derives from enhanced GlyR signaling can be achieved by (partial) GlyT2‐inhibition, besides GlyT1‐blockade or exogenous delivery of glycine. *Mesencephalic origin of glycinergic projections presented in abstract by Konar et al. (2022).

In contrast to GlyT1, GlyT2 is exclusively expressed within the glycinergic synapse, at the end‐terminal of glycinergic neurons where it is juxtaposed to postsynaptic GlyRs and enables refilling of glycine‐containing vesicles (Eulenburg et al. 2005). Canonically, it has been postulated that GlyT2 is mainly expressed in caudal regions of the CNS, that is, the spinal cord, brain stem and cerebellum (APA 2013; Eulenburg et al. 2005; Zeilhofer et al. 2005). Interestingly, convergent data from a recent study that combined immunoblotting, immunohistochemistry and electrophysiological studies demonstrated that GlyT2 and glycinergic currents are also present within the nAc (Muñoz et al. 2018), thus inferring that GlyR‐induced effects on the reward circuitry may involve both intra‐ and extrasynaptic GlyRs. A schematic overview of hypothesized glycinergic neurotransmission in the nAc is provided in Figure 1. In the context of accumbal DA output or addiction, pharmacological interventions that target the GlyT2 have hitherto not been explored. However, different classes of GlyT2‐inhibitors have been extensively evaluated in animal models of neuropathic and inflammatory pain, based on the rationale that increased synaptic glycine levels enhance glycinergic neurotransmission in the dorsal horn of the spinal cord, ultimately alleviating pain (Cioffi 2021). Lately, the field has shifted focus from full and irreversible to partial and reversible GlyT2‐inhibitors as the former compounds are associated with substantial side effects in primarily mice but also rats, presumably due to chronic GlyT2‐blockade gradually depleting glycine‐containing vesicles of their content (Cioffi 2021). In contrast, partial GlyT2‐inhibitors have demonstrated analgesic effects devoid of adverse events in both rats (Cioffi 2021) and in a Phase III clinical trial for post‐surgical pain (Lee et al. 2025), inferring that partial GlyT2‐blockade elevates synaptic glycine levels while still allowing for a small level of glycine re‐uptake and refilling of vesicles (Cioffi 2021).

In light of the preclinical rationale for enhanced accumbal GlyR activation as a mechanism to raise and/or restore DA levels in the nAc, thereby reducing alcohol intake, combined with recently presented evidence for synaptic glycinergic activity in the nAc, this study sought to explore whether GlyT2‐inhibition also alters accumbal glycine and DA output, as well as alcohol intake in rats. Consequently, the effects of systemic administration of two partial GlyT2‐inhibitors with distinct characteristics, N‐Arachidonyl‐Glycine (NAGly) and Opiranserin, and the full GlyT2‐inhibitor Org 25,543, were explored with respect to their effects on extracellular glycine and DA levels using in vivo microdialysis in male Wistar rats. Further, the effects of NAGly treatment were examined in a voluntary alcohol consumption model in rats. The effects of combined blockade of GlyT1 and GlyT2 on extracellular glycine and DA output in the rat nAc were also examined. Finally, immunohistochemistry was used to visualize GlyT2 and GlyR in the nAc.

## 2 Materials and Methods

### 2.1 Animals

**FIGURE 2:**
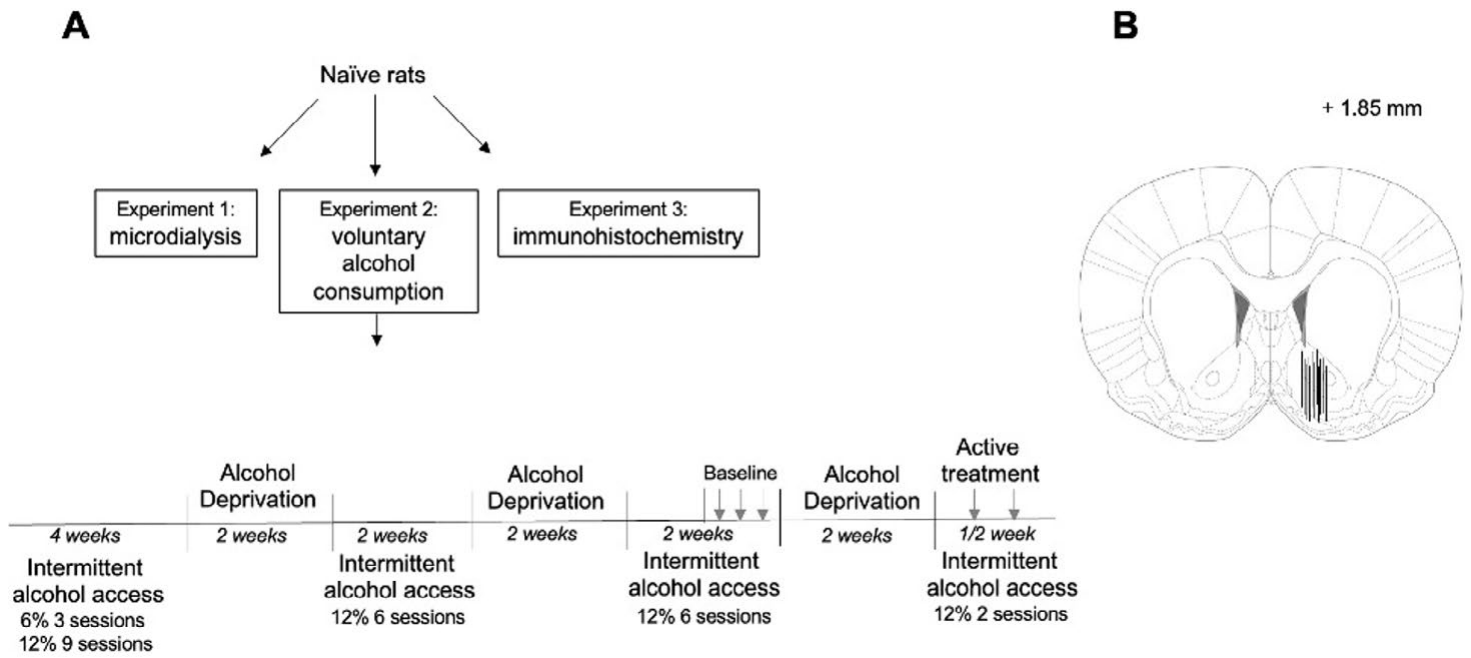
Overview of experimental design and verification of probe placement. (A) Schematic overview of the experimental design. In a first set of experiments, in vivo microdialysis was used to examine effects on accumbal glycine and dopamine levels. Thereafter, effects on voluntary alcohol intake and the alcohol deprivation effect were assessed in a new group of rats, using a paradigm that entailed repeated cycles of alcohol deprivation prior to active treatment. Gray arrows indicate systemic injections with vehicle at baseline and NAGly or vehicle during the active treatment phase. Finally, brains from a new group of rats were obtained for immunohistochemistry on brain sections at the level of the nAc. (B) Location of 17 representative microdialysis probes in the nAc. Black bars indicate the active space of the probe and targets the nAc core–shell borderline region. Distance from bregma is indicated on the right side of the figure.

A schematic overview of all experiments is provided in Figure 2A. For in vivo microdialysis and immunohistochemistry studies, a total number of 114 male Wistar rats weighing 260–280 g upon arrival were supplied by Envigo (Netherlands). Rats were housed four to a cage prior to surgery and single‐housed after surgery with regular light/dark conditions with lights on at 07:00 a.m. and off at 07:00 p.m., constant room temperature of 20°C–22°C and humidity of 50%–65%. For the alcohol consumption study, 50 male Wistar rats weighing 260–280 g upon arrival were supplied by Envigo (Netherlands). During the first week, rats were group‐housed under controlled environmental conditions with a regular light–dark cycle as above. Thereafter, rats were single‐housed with a reversed light–dark cycle, that is, lights off at 10:00 a.m. and lights on 10:00 p.m. Before initiation of experimental procedures, all animals were allowed to adapt to the novel environment at the animal facility for at least 1 week. All rats had free access to tap water and standard rat feed (Haklan Teklad, England). Based on a previous study in rats (Olsson et al. 2023) in which pharmacotherapy aimed at GlyR signaling (1) abolished the ADE in a voluntary alcohol consumption study and (2) raised accumbal DA levels as measured by in vivo microdialysis, a power calculation suggested that a sample size of *n* = 48 in the voluntary alcohol consumption study versus *n* = 10 (in each treatment arm) in the microdialysis study would yield a power of 0.80 at an alpha of 0.05 (Faul et al. 2007). The experiments were approved by the Ethics Committee for Animal Experiments, Gothenburg, Sweden (ethical approval reference number 2401‐19, 3095‐20 and 25‐70).

### 2.2 Drugs

NAGly (Enzo Biochem Inc., USA) was dissolved in 0.01 M phosphate‐buffered saline (PBS) (Sigma‐Aldrich, Sweden) to a desired dose of 1, 5, 10 or 20 mg/kg and administered i.p. The doses applied here were primarily based on doses used in pain models in rats, that is, when studying drug effects that presumably stem from interference with infratentorially located GlyT2 and GlyRs, as studies in supratentorial brain regions (e.g., in vivo microdialysis) and alcohol‐related, behavioral studies on GlyT2‐inhibitors, including NAGly, are lacking. It has previously been reported that NAGly in doses spanning from 70 to 700 nmol (approx. 70 μg/kg—0.7 mg/kg) administered i.t. (Succar et al. 2007; Vuong et al. 2008) and a dose of 40 mg/kg p.o. dissolved in peanut oil (Burstein 1999), produced analgesic effects without being associated with adverse side effects. Opiranserin/VVZ‐149 (MedChemTronica, Sweden) was dissolved in 0.01 M PBS to a desired dose of 20 mg/kg (administered i.p.), in line with a previous study in which this dose was effective in reducing allodynia and pain‐related behavior in rats (Pang et al. 2012). Org 25,543 (Sigma‐Aldrich, Sweden) was dissolved in 5% v/v dimethyl sulfoxide (DMSO) in 0.01 M PBS to a desired dose of 10 and 15 mg/kg (administered i.p.). A previous microdialysis study has shown that rats perfused with either Org 25,543 or the GlyT1‐inhibitor Org 24,598 into the dorsal spinal cord in a dose of 10 μm presented a substantial (threefold) and equal elevation in spinal glycine levels (Whitehead et al. 2004). As systemic treatment with Org 24,598 in a dose of 6 and 9 mg/kg have been demonstrated to produce a twofold elevation in accumbal glycine levels (Olsson et al. 2023), it can be inferred that a similar (or slightly higher) dose would also be applicable for systemic treatment with Org 25,543, albeit ratios for the abundance of GlyT1 and GlyT2 probably differ between the spinal cord and the nAc. In rat models of pain, nociceptive effects were achieved for doses spanning between 4 and 30 mg/kg i.p., (Mohammadzadeh et al. 2021; Mostyn et al. 2019), although the higher dose was associated with substantial side effects, thus constituting an upper dose limit (Mostyn et al. 2019). Bitopertin (Ambeed) was dissolved in 1% v/v Tween 80 in 0.01 M PBS to a desired dose of 10 mg/kg and administered via oral gavage. This dose has been shown to yield a twofold increase in striatal extracellular glycine levels (Alberati et al. 2012), that is, to an extent that has previously been associated with a GlyR‐mediated DA elevation in the nAc (Söderpalm et al. 2017). All compounds were administered using an injection volume of 5 mL/kg. Ethanol (95%; Kemetyl AB, Sweden) was diluted in tap water to a concentration of 6% and 12% (v/v), in line with alcohol concentrations chosen in previous alcohol consumption studies that explore the glycinergic system (Olsson et al. 2021, 2023) and other pharmacological treatment targets for AUD (Loftén et al. 2025; Söderpalm et al. 2020). The vehicle treatment consisted of either PBS i.p., 5% DMSO in PBS i.p. or 1% Tween 80 in PBS p.o., in order to match the solvent medium for respective active treatments.

### 2.3 Microdialysis Study

Two days prior to the microdialysis experiment, probe placement surgery was performed. The rats were anesthetized with 4% isoflurane (Baxter, Sweden) and mounted onto a stereotactic instrument (David Kopf Instruments, USA). Thereafter, the rats were placed on a heating pad, received 2 mL 0.9% saline, 1 mg/kg Metacam (Boehringer Ingelheim, Sweden) s.c., and Marcain (bupivacaine, AstraZeneca, Sweden) infiltrated along the surgical incision to prevent hypothermia and dehydration and provide analgesia during surgery. Three holes were drilled in the skull in which two anchoring screws and an I‐shaped, custom‐made probe with a semi‐permeable membrane (cut‐off 20 kDa) and an active space comprising 2 mm were placed. The probe was gently lowered into the nAc core–shell borderline region (A/P: +1.85, M/L: −1.4, D/V: −7.8 mm relative to bregma and dura), coordinates from Paxinos and Watson (2007), as this sub‐region of the nAc has previously been associated with alcohol‐induced DA elevation (Howard et al. 2009). Harvard cement (Dental AB, Sweden) was used to fix the probe and anchoring screws to the skull.

In the microdialysis experiment, rats were connected to a microinfusion pump (U‐864 Syringe Pump, AgnTho's, Sweden) via a swivel, which allowed the animal to move around freely in its cage. The probes were perfused with Ringer's solution during 2 h prior to sampling of microdialysate content, to achieve equilibrium between the perfusate and extracellular fluid. Thereafter, dialysate samples of 40 μL were collected every 20 min, using an infusion rate of 2 μL/min. The baseline in DA levels for each animal consisted of the first three, consecutively sampled dialysates and was set to 100%. Animals were excluded if fluctuations at baseline exceeded 10%. All designated treatments were administered i.p. or via oral gavage at timepoint 0 min and dialysate samples were collected for another 180 min. Microdialysate samples were analyzed online for DA and the remainder of the dialysate was preserved in sodium azide and stored at a cold temperature for later analyses of glycine content. DA and glycine content in the dialysate were separated and quantified using a high‐performance liquid chromatography (HPLC) system coupled to electrochemical (DA) or fluorescent (glycine) detection as previously described (Ulenius et al. 2020). In order to identify DA and glycine peaks in the chromatogram and to quantify each analyte, external standards containing 3.25 nm of DA versus 500 and 1000 nm of glycine were used, respectively. At the end of the experiment, rats were sacrificed, and a vibroslicer (Campden Instruments Ltd., USA) was used to cut brains into thin slices so that a correct probe placement could be verified with the naked eye. Rats with incorrect probe placement or substantial hemorrhage were excluded (*n* = 11). Probe placement is illustrated in Figure 2B.

### 2.4 Voluntary Alcohol Consumption Study

Throughout the experiment, all rats had free access to tap water and standard rat feed, were monitored daily and weighed on a regular basis (Figure 6A). Further, rats were evaluated for alcohol and water intake during 14.5 weeks with intermittent alcohol access. Rats were presented with an alcohol concentration of 6% (v/v) during the first three sessions followed by 12% (v/v) for the remainder of the experiment. During the first 4 weeks, rats had intermittent (3 sessions/week) access to alcohol. Thereafter, rats were placed on a paradigm that comprised 2 weeks of alcohol deprivation followed by 2 weeks of intermittent (3 sessions/week) alcohol access. The protocol was repeated for three cycles in order to induce an Alcohol Deprivation Effect (ADE), ascribed to reflect a modality of AUD, that is, loss of control in limiting alcohol intake (Spanagel and Hölter 1999). Following the third cycle of alcohol deprivation, intermittent alcohol access was reinstated for 2 days. All rats were divided into treatment groups based on their average alcohol consumption and received either active treatment (NAGly 10 mg/kg i.p.) or vehicle injections on days that alcohol was provided. These procedures allowed for evaluation of ensuing drug effects on the ADE, which has a high predictive validity for clinical effect (Spanagel and Hölter 2000). In order to minimize the influence of stress brought by the injection on alcohol consumption, rats received preparatory vehicle injections in volumes equal to active treatment also during the baseline week, that is, the last week with intermittent alcohol access that preceded the third alcohol deprivation period. All injections were administered 30 min prior to access to alcohol and water, in similarity to a previously performed study in which GlyT1‐inhibitors were evaluated on alcohol intake (Olsson et al. 2023).

### 2.5 Immunohistochemistry

Rats were deeply anesthetized with Alfatal vet. (100 mg/mL Apoteket AB, Sweden, 350 mg/kg i.p.) and perfused transcardially with 0.9% NaCl at room temperature, buffer (116 mm NaCl, 5.4 mm KCl, 1.6 mm MgCl2, 0.4 mm MgSO4, 1.3 mm NaH2PO4, 26 mm NaHCO3, 5.5 mm glucose), followed by 4% ice‐cold paraformaldehyde solution (pH 7.4) before the brains were removed. The brains were post‐fixed in paraformaldehyde solution for 4 h and cryo‐protected via immersion in 30% sucrose solution. Thereafter, brain sections (30 μm) at the level of the nAc were cut on a freezing sliding microtome (Bright instruments, UK) and stored free‐floating at −20°C in anti‐freeze solution (30% ethylene glycol and 20% glycerol in 1X PBS). Following washing, sections were incubated in blocking solution (1% bovine serum albumin and 0.3% Triton X‐100 in 1× PBS) for 45 min and transferred to primary antibody solution containing polyclonal rabbit anti‐GlyT2 (272003, Synaptic Systems, 1:5000 for combination with chicken anti‐MAP2, 1:1000 for combination with mouse anti‐GlyR) and polyclonal chicken anti‐MAP2 (188006, Synaptic Systems, 1:200) or mouse anti‐GlyR (146011, Synaptic Systems, 1:250), and incubated overnight at room temperature. When sections were co‐stained for GlyT2 and MAP2, they were rinsed and immersed in 0.3% H_2_O_2_ for 30 min to block endogenous peroxidase activity. Following additional rinsing, sections were then incubated in secondary antibodies (for GlyT2: HRP‐conjugated donkey anti‐rabbit, 1:2000, Invitrogen; for MAP2: Alexa Fluor 555‐conjugated donkey anti‐chicken, 1:1000, Jackson ImmunoResearch), for 1 h at room temperature. After rinsing, sections were incubated in Alexa 488‐conjugated tyramide (1:200, Invitrogen SuperBoost kit) for 10 min. When sections were co‐stained for GlyT2 and GlyR, sections were rinsed and incubated in secondary antibodies (for GlyT2: Alexa Fluor 488‐conjugated donkey anti‐rabbit, 1:500, Invitrogen; for GlyR: Alexa Fluor 647‐conjugated donkey anti‐mouse, 1:500, Invitrogen) for 1 h at room temperature. After additional rinsing, sections were mounted onto SuperFrost Plus slides, air‐dried, and coverslipped using ProLong Gold Antifade mounting medium (Invitrogen).

### 2.6 Imaging

Low magnification images were obtained using an epi‐fluorescent Axio Observer Z1 widefield microscope equipped with a 10× or 20× objective and a 14‐bit AxioCam 506 mono camera (Zeiss). Tile scans were captured to cover the entire area of interest. Shading correction was applied and images were stitched using the Zen Blue software (v. 2.6). High magnification images were instead captured using a Zeiss LSM 700 Inverted confocal microscope (Zeiss, Jena, Germany) using a 40×/1.3 oil objective. Tile scans and Z‐stacks were acquired and stitched using Zen Blue software, and the raw images were imported into FIJI (NIH) where maximum intensity projections were made. Brightness and contrast were adjusted equally across the channels for all images. Resizing of images and cropping were performed in Affinity Photo (v. 2.6.0), and the photos were annotated and assembled into panels using Affinity Designer (v. 2.6.0).

### 2.7 Statistics

Alterations in alcohol consumption and microdialysate content over time were evaluated by a two‐way analysis of variance (ANOVA) with repeated measures (*t* = 0–180 min for in vivo microdialysis, *t* = day 0–2 for the voluntary alcohol consumption study), whereas comparisons of area under the curve (AUC) data were performed using a one‐way ANOVA, followed by Tukey's or Dunnett's post hoc when applicable. Alcohol intake prior to and after the alcohol deprivation period was evaluated with a paired *t*‐test for effects within a treatment group and with a one‐way ANOVA for effects between the treatment groups. All data are presented as mean ± SEM. A probability value (*p*) < 0.05 was considered statistically significant. Statistical analyses were performed using GraphPad Prism version 10.4.1 for Mac OS (GraphPad Software Inc., USA).

## 3 Results

**FIGURE 3:**
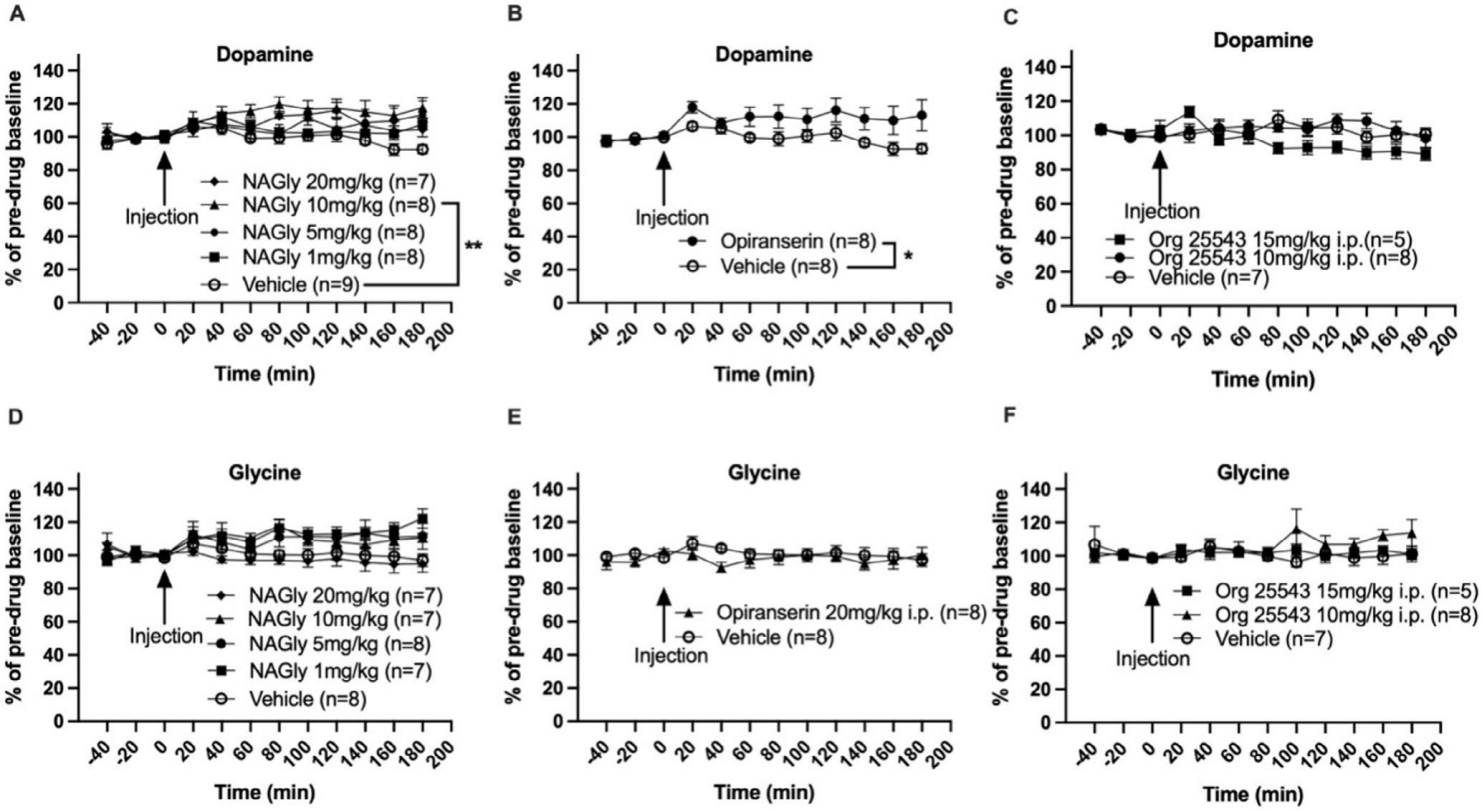
Effects of three different GlyT2‐inhibitors with distinct characteristics on accumbal DA and glycine levels. (A) Systemic treatment with NAGly in a dose of 10 mg/kg i.p. slightly raised nAc DA levels while the other doses examined (1, 5, 20 mg/kg) did not significantly alter DA output. (B) Systemic treatment with Opiranserin 20 mg/kg i.p. produced a minor, albeit significant elevation in accumbal DA levels. (C) Systemic treatment with Org 25,543 10 and 15 mg/kg i.p. did not significantly alter accumbal DA levels, albeit a significant time × treatment effect was observed. There were no effects on extracellular glycine following systemic treatment with neither of the partial GlyT2‐inhibitors (D) NAGly, (E) Opiranserin or (F) the full GlyT2‐inhibitor Org 25,543. Analyses were performed for *t* = 0–180 min. Shown are mean values ± SEM, *n* = number of rats, *p < 0.05, **p < 0.01.

In a first set of experiments, three GlyT2‐inhibitors with distinct characteristics were evaluated in a microdialysis experiment. A two‐way‐ANOVA_*t* = 0–180 min_ revealed that the partial GlyT2‐inhibitor NAGly slightly raised extracellular DA levels in the nAc as compared to vehicle treatment (treatment effect *F*_(4,34)_ = 2.83, *p* = 0.040, time effect *F*_(9,306)_ = 3.31 *p* < 0.001, interaction term *F*_(36,306)_ = 1.75, *p* = 0.007, Figure 3A). Following Dunnett's multiple comparisons test, a dose of 10 mg/kg i.p. remained significant (*p* = 0.008), whereas other doses that were evaluated (1, 5 and 20 mg/kg) did not significantly alter DA output. Also the combined partial GlyT2‐inhibitor, 5HT2A‐ and P2X3‐antagonist Opiranserin slightly but significantly raised accumbal DA levels (treatment effect *F*_(1,14)_ = 5.37, *p* = 0.036, time effect *F*_(9,126)_ = 1.68 *p* = 0.100, interaction term *F*_(9,126)_ = 1.11, *p* = 0.364, Figure 3B), but to a lesser extent as compared to NAGly treatment. The full GlyT2‐inhibitor Org 25,543 produced a significant time × treatment interaction on nAc DA output (*F*_(18,153)_ = 2.14, *p* = 0.007) but no treatment nor time effect per se (Figure 3C). In contrast to what was expected, none of the GlyT2‐inhibitors produced any effects on extracellular glycine levels in the nAc (Figure 3D–F), as measured by in vivo microdialysis.

**FIGURE 4:**
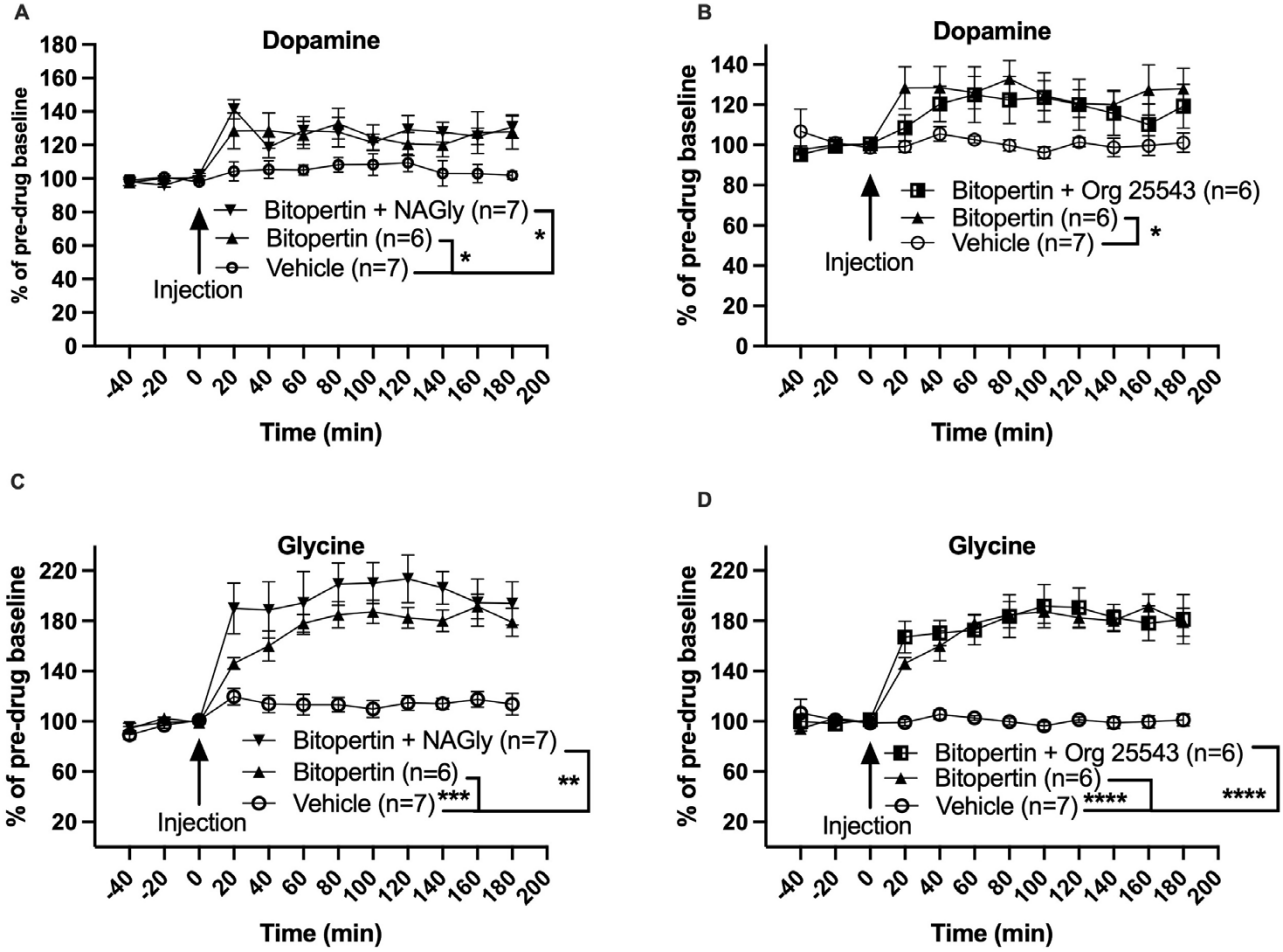
Effects of combined GlyT1‐ and GlyT2‐inhibition on accumbal DA and glycine levels. (A) Systemic treatment with the reversible GlyT1‐inhibitor Bitopertin (10 mg/kg p.o.) in monotherapy or combined with the partial GlyT2‐inhibitor NAGly (10 mg/kg i.p.) raised accumbal DA levels. However, combined treatment with Bitopertin and NAGly did not produce any additive effects on accumbal DA output. (B) Similarly, combined treatment with Bitopertin and the full GlyT2‐inhibitor Org 25,543 did not yield any additive effects on accumbal DA levels. (C) Accumbal glycine levels were robustly raised following systemic treatment with bitopertin and following combined treatment with bitopertin and NAGly. Although a visual trend for additive effects on accumbal glycine levels could be discerned, these effects were not statistically significant. (D) Further, no additive effects on accumbal glycine levels were observed following combined systemic treatment with Bitopertin and Org 25,543. Analyses were performed for *t* = 0–180 min. Shown are mean values ± SEM, *n* = number of rats, *p < 0.05, **p < 0.01, ***p < 0.001, ****p < 0.0001.

Next, the reversible GlyT1‐inhibitor Bitopertin was co‐administered with NAGly, in order to evaluate whether the drugs combined would produce additive effects on accumbal DA and glycine output. As expected, monotherapy with Bitopertin in a dose of 10 mg/kg p.o. and combined treatment with Bitopertin and NAGly robustly raised nAc DA levels (two‐way ANOVA_*t* = 0–180 min_; treatment effect *F*_(2,16)_ = 7.28, *p* = 0.006, time effect *F*_(9,144)_ = 6.81 *p* < 0.0001, interaction term *F*_(18,144)_ = 1.70, *p* = 0.046, Bitopertin vs. vehicle *p* = 0.011, Bitopertin + NAGly vs. vehicle *p* = 0.016). However, no additive effects on DA output were observed for the doses evaluated (Figure 4A). In similarity to the effects observed for Bitopertin + NAGly, combined treatment with Bitopertin and the full GlyT2‐inhibitor Org 25,543 did not produce any additive effects on DA output (Figure 4B). Upon evaluation of glycine levels, bitopertin alone and in combination with NAGly produced a pronounced elevation in extracellular, accumbal glycine levels (treatment effect *F*_(2,16)_ = 15.0, *p* = 0.0002, time effect *F*_(9,144)_ = 25.2 *p* < 0.0001, interaction term *F*_(18,144)_ = 5.38, *p* < 0.0001, Bitopertin vs. vehicle *p* = 0.005, Bitopertin + NAGly vs. vehicle *p* = 0.0002), while the two treatment regimens did not significantly differ, (Figure 4C). Similarly, combined treatment with Bitopertin and Org 25,543 did not reveal any additive effects on glycine levels as compared to monotherapy with Bitopertin (Figure 4D).

**FIGURE 5:**
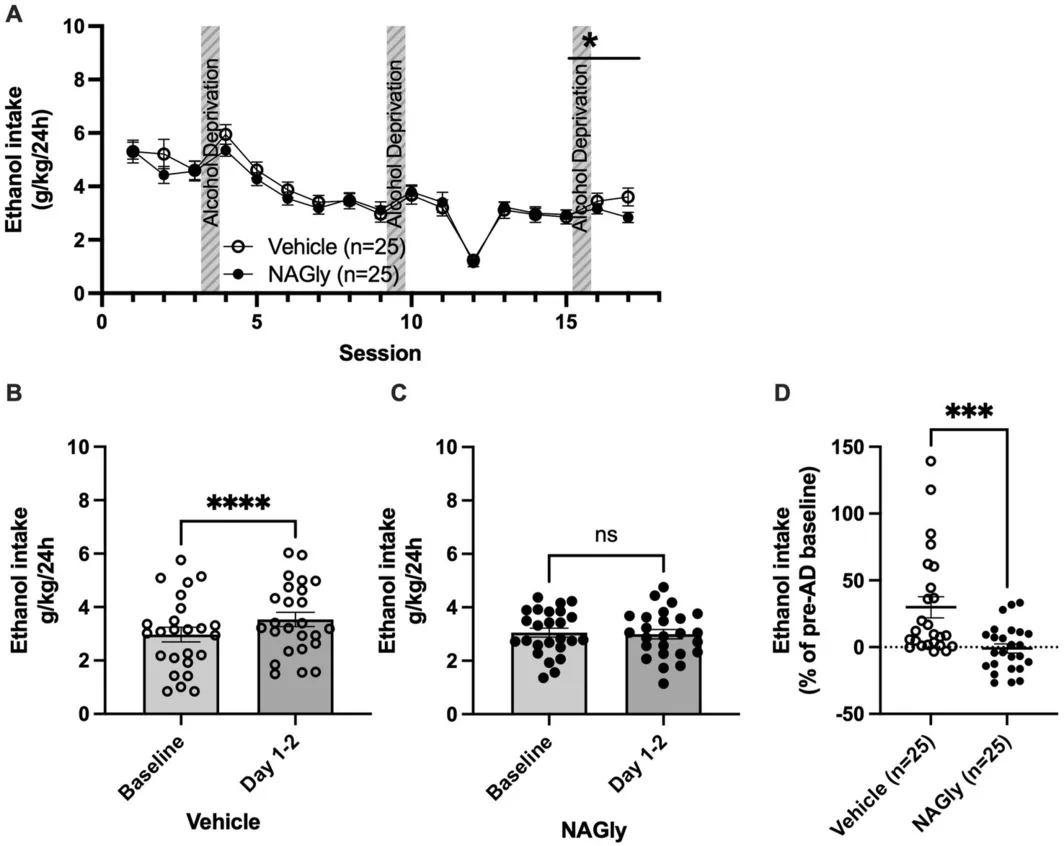
Effects of NAGly, on voluntary alcohol intake and the alcohol deprivation effect (ADE). (A) Time course graph depicting alcohol intake over time in a drinking paradigm that entails intermittent alcohol access and repeated cycles with alcohol deprivation whereafter rats are co‐exposed to alcohol and treatment with NAGly or vehicle for 2 days. Following the first and second alcohol deprivation period, no alterations in alcohol intake, as compared to baseline, was observed for the full sample. (B) Vehicle‐treated rats presented an ADE, that is, a transient increase in alcohol intake as compared to baseline, on the 2 days of treatment that followed the third alcohol deprivation period, while (C) this effect was abolished in NAGly‐treated rats. (D) Upon comparison of the percental change in alcohol intake after (mean of two values) as compared to before (mean of three values) the alcohol deprivation period, NAGly‐treated rats presented a lower relative alcohol intake. Similarly, a two‐way ANOVA yielded a significant time × treatment interaction term for data presented in (A), suggesting that the treatment affected the ADE. Throughout the experiment, rats had intermittent access to 12% alcohol (24 h‐sessions 3d/week) except during the indicated weeks with alcohol deprivation. Shown are mean values ± SEM, *n* = number of rats, *p < 0.05, **p < 0.01, ***p < 0.001.

**FIGURE 6:**
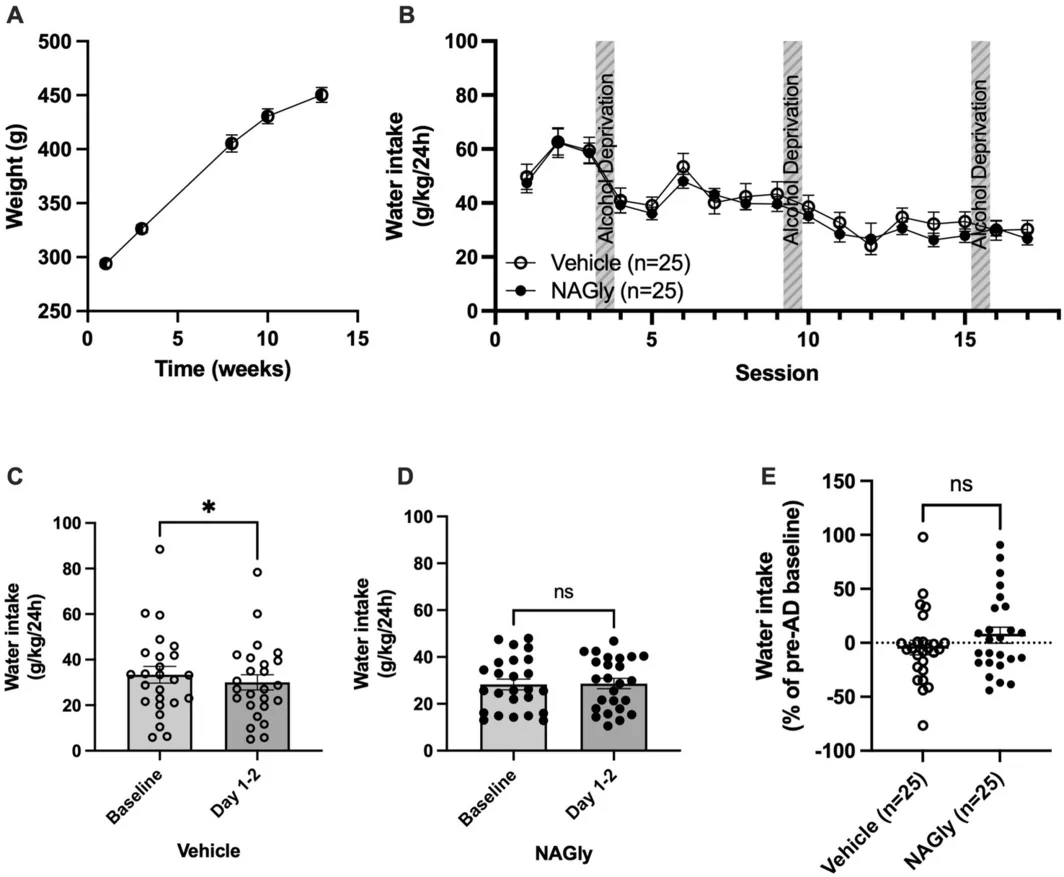
Weight gain and effects of NAGly on water intake. (A) Rats presented a stable weight gain throughout the experiment. (B) Time course graph depicting water intake in a voluntary alcohol consumption paradigm that entailed constant access to water and intermittent access to or deprivation from alcohol. Throughout the experiment, but prior to drug administration with NAGly vs. vehicle, water intake was not significantly altered. Following the third and last alcohol deprivation period, (C) vehicle‐treated rats but not (D) NAGly‐treated rats presented a decreased water intake as compared to baseline. (E) Upon comparison of the percental change in water intake after (mean of two values) as compared to before (mean of three values) the alcohol deprivation period, no differences between the treatment groups were observed. Shown are mean values ± SEM, *n* = number of rats, *p < 0.05.

In the next experiment, NAGly in a dose of 10 mg/kg was chosen for further evaluation in a voluntary alcohol consumption study with 4 weeks of intermittent alcohol access followed by three cycles with 2 weeks of alcohol deprivation flanked by 2 weeks with intermittent alcohol access. NAGly was chosen over Opiranserin as its DA‐elevating effect appeared numerically larger in the microdialysis study and as NAGly more selectively target the glycinergic system. The average daily alcohol intake was monitored throughout the experiment (Figure 5A), that in total spanned over 15 weeks, and was on average 3.7 g/kg/day. The baseline drinking level was defined as the average alcohol intake for each rat during the last three sessions that preceded each alcohol deprivation period. The alcohol intake in the full sample was not altered as compared to baseline after the first and second alcohol deprivation period. Following the third and last alcohol deprivation period, intermittent alcohol was reinstated for 2 days. Rats were divided into two treatment groups that presented equal alcohol intake at baseline (*t*_(48)_ = 0.255, *p* = 0.800) and received either active treatment (NAGly 10 mg/kg i.p) or vehicle (PBS i.p. in equal injection volumes) on the 2 days with alcohol access. Increased alcohol intake, that is, an ADE, was evident in the vehicle treatment group on the first and second day (mean of two values) with reinstated alcohol access that followed the last alcohol deprivation period (*t*_(24)_ = 4.736, *p* < 0.0001 Figure 5B). In contrast, NAGly treatment appeared to block the ADE as no within‐group effects were evident in a paired *t*‐test (*t*_(24)_ = 0.596, *p* = 0.557, Figure 5C) and as a two‐way‐ANOVA_*t* = day 0–2_ revealed a significant interaction between treatment and time (treatment effect *F*_(1,48)_ = 1.14, *p* = 0.29, time effect *F*_(2,96)_ = 2.12 *p* = 0.126, interaction term *F*_(2,96)_ = 4.54, *p* = 0.013, Figure 5A). Further in line with these results, NAGly‐treated rats presented a lower, relative alcohol intake upon comparison of the percental change in alcohol after (mean of two values) as compared to before (mean of three values) the alcohol deprivation period (*t*_(48)_ = 3.57, *p* = 0.0008, Figure 5D). Animal weight (Figure 6A) and water intake (Figure 6B) were monitored throughout the experiment, and all rats presented a stable weight gain. A two‐way‐ANOVA_*t* = day 0–2_ revealed no alterations in water intake over time or differences between treatment groups (Figure 6B). In contrast to the effects observed on alcohol consumption, water intake following alcohol deprivation was decreased in the vehicle‐treated group (*t*_(24)_ = 2.083, *p* = 0.048) but not altered in the NAGly‐treated group (Figure 6C). There were no differences in the relative change in water intake (Figure 6D).

**FIGURE 7:**
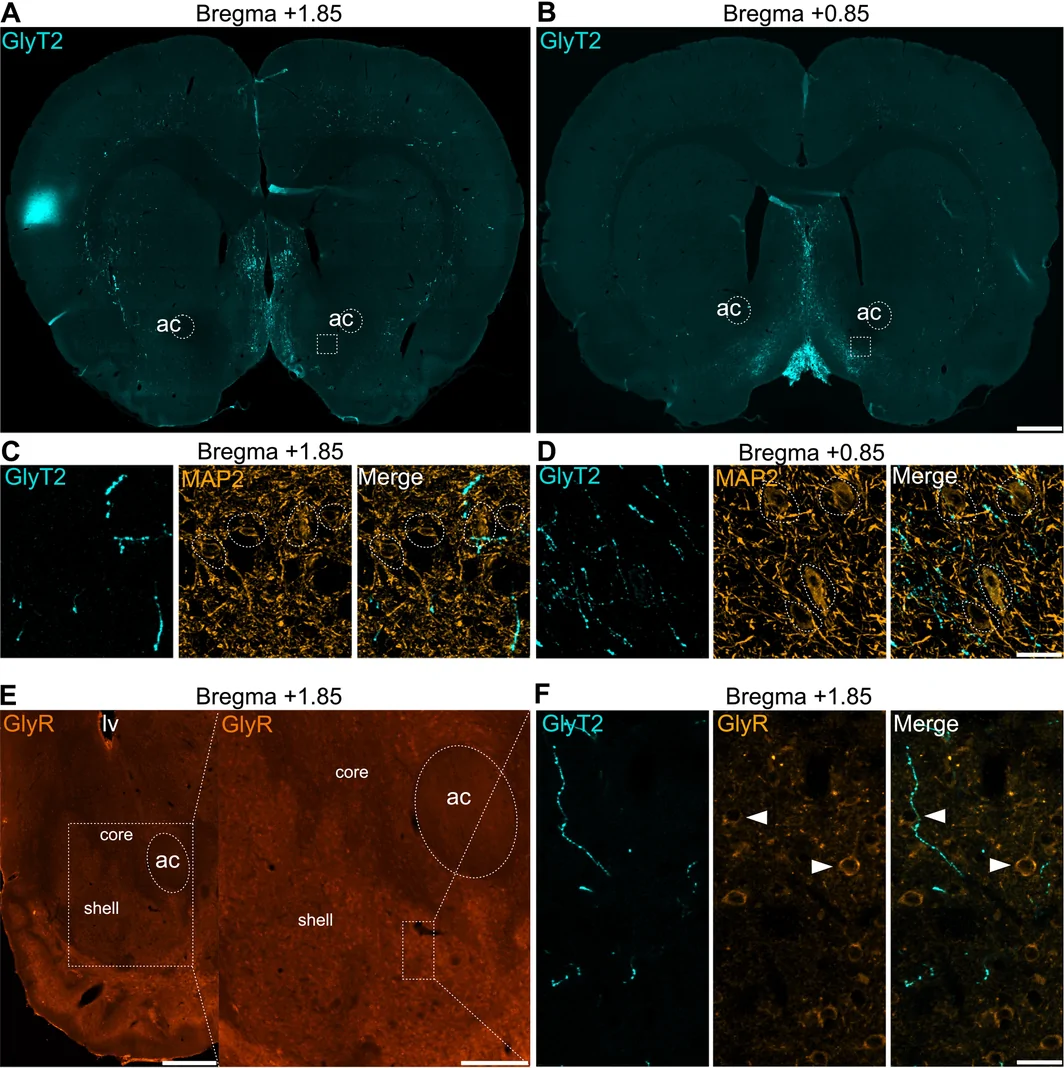
Presence of GlyT2 and GlyR in the nAc. (A) Coronal brain slices at the level of bregma +1.85 mm showed immunoreactivity for GlyT2‐fibers (cyan), displaying an axonal distribution pattern in lateral aspects of the nAc, the dorsolatereral striatum and in cortical regions of the forebrain. GlyT2‐fibers were more abundant in the medial and lateral septum and basal forebrain regions, that is, the VDB. Scattered immunoreactivity for GlyT2‐fibers was observed in medial aspects of the nAc core–shell border region targeted in the microdialysis study. (B) In more caudal coronal brain slices, at bregma +0.85 mm, GlyT2‐fibers were more abundant in the medial parts of the nAc shell, in the medial septum, in basal forebrain regions, that is, VDB and the medial forebrain bundle. (C, D) Confocal microphotographs from coronal brain slices at bregma +1.85 mm (C) and bregma +0.85 (D), showing immunoreactivity in the nAc for GlyT2 (cyan) and the neuronal soma/dendritic marker MAP2 (orange), where the neuronal somas are circled for clarity. The merged image indicates the presence of axonal GlyT2‐ fibers in proximity to MAP2‐expressing neuronal somas. (E) Coronal brain section imaged at the level of bregma +1.85 mm, displaying immunoreactivity for GlyR (orange) that appeared to be more pronounced in the shell and core–shell border region of the nAc as compared to the nAc core. Boxed region in the left image is magnified to the right. (F) Confocal microphotograps displaying GlyT2 immunoreactivity (cyan) and GlyR immunoreactivity (orange). While GlyT2 displayed an axonal distribution pattern, GlyR‐labelling was instead associated with a membrane distribution pattern. White arrows indicate two of the GlyR‐expressing cells. The merged image indicates the presence of axonal GlyT2‐fibers in proximity to GlyR‐expressing cells bodies. Scale bars represent 1 mm in coronal images (A) and (B), 500 and 250 μm in (E) and 25 μm in high magnification images (C), (D), (F). *The high magnification images at the level of bregma +1.85 in (C) were obtained from an adjacent brain section (at bregma +1.85) to the coronal brain section presented in (A). ac, anterior commissure; lv, lateral ventricle.

Immunohistochemistry studies were performed on coronal brain slices collected from alcohol‐naïve rats. At the anterio‐posterior level of bregma +1.85 mm, that is, the coordinates applied for registering accumbal DA and glycine output in the microdialysis study, the presence of GlyT2‐expressing neuronal fibers was scarce in the medial core–shell border region of nAc and slightly more abundant in lateral aspects of the nAc shell (Figure 7A). More caudally, at the level of bregma +0.85 mm, GlyT2‐expressing axonal fibers were slightly more present in medial parts of the nAc shell as shown on coronal brain slices in Figure 7B and in the confocal microphotographs (bregma +1.85 mm in Figure 7C, bregma +0.85 mm in Figure 7D) where, at both levels, immunoreactivity was shown for GlyT2 (cyan) and the neuronal soma/dendritic marker MAP2 (orange). The merged microphotographs indicate that the GlyT2‐expressing fibers, expressed in a pattern suggestive of axonal origin, are positioned in proximity to MAP2‐expressing neuronal somas. Moreover, GlyT2‐fibers were highly abundant in septal and basal forebrain regions, that is, medial and lateral septum, vertical limb of the diagonal band of Broca (VLB) and the medial forebrain bundle and displayed an intermediate expression in cortical regions of the forebrain and in the dorsolateral striatum (Figure 7A,B). Furthermore, the presence of GlyR immunoreactivity (orange) was confirmed in medial aspects of the nAc (bregma +1.85 mm) (Figure 7E), with a higher abundance in the shell and core–shell border region as compared to the nAc core. Confocal microphotographs (Figure 7F) revealed a distribution pattern consistent with plasma membrane localization. In addition, GlyR‐expressing cell bodies were detected in close proximity to GlyT2‐containing axonal fibers (Figure 7F).

## 4 Discussion

The GlyT2 has recently been established to be present in the nAc and this study explored its role, alongside GlyT1, in regulating basal accumbal DA levels and voluntary alcohol intake in rats. Notably, systemic treatment with two structurally different, partial GlyT2‐inhibitors, NAGly and Opiranserin, demonstrated a slight, albeit significant elevation in extracellular DA levels in the nAc. This effect was less pronounced as compared to partial GlyT1‐inhibition using Bitopertin. Furthermore, in rats exposed to intermittent access to alcohol with repeated cycles of alcohol deprivation, treatment with NAGly reduced the relative alcohol intake and abolished the ADE, probably as a result of its indirect interference with GlyR and DA output in the nAc. In line with the results from the in vivo experiments, the presence of GlyT2‐expressing axonal fibers in distinct parts of the nAc was confirmed and a high concentration of GlyT2‐expressing fibers in brain regions of relevance in reward, for example, the lateral septum and medial forebrain bundle, was demonstrated.

In a series of studies, our research group has previously proposed a mechanism for how accumbal GlyRs regulate basal and alcohol‐induced DA output in the nAc by disinhibiting DA neurons projecting between VTA and nAc (Söderpalm et al. 2017). These events have been causally linked to the level of voluntary alcohol consumption in rats in studies that implemented monitoring of extracellular glycine and DA levels in the nAc and alcohol consumption within the same animal (Söderpalm et al. 2017). It has been theorized that enhanced GlyR‐activity and ensuing effects on DA output may reduce alcohol intake by (I) compensating for pre‐existing or alcohol‐induced deficits in mesoaccumbal DA transmission and (II) inducing a transient desensitization of accumbal GlyRs so that alcohol, when co‐administered with direct or indirect GlyR agonists such as glycine or GlyT1‐inhibitors, cannot elicit any further DA release, thereby preventing negative and positive reinforcement to consume alcohol. Findings from independent research groups also support the notion that enhanced GlyR activity plays a protective role for regulating alcohol intake as, for example, three structurally and functionally different GlyT1‐inhibitors were demonstrated to reduce alcohol‐seeking and to abolish relapse‐like drinking in rats (Vengeliene et al. 2018), and as overexpression of alcohol‐sensitive GlyRα1‐subunits in accumbal, GABA‐ergic neurons reduced binge drinking in mice (Araya et al. 2023).

In the present study, accumbal DA levels were slightly raised following systemic treatment with NAGly, and to a lesser extent by Opiranserin. A possible mechanism for this event could be that GlyT2s in the nAc are targeted, yielding increased glycine concentrations in the synaptic cleft and enhanced activation of synaptic GlyRs in the nAc, whereby mesoaccumbal DA projections are disinhibited. However, results from the in vivo microdialysis experiments, reflecting extracellular levels of, for example, glycine, did not display any alterations in accumbal glycine levels following neither full nor partial GlyT2‐inhibition. These results suggest that only negligible amounts of synaptic glycine have diffused into the extracellular space or that the time resolution for HPLC (20 min) is too low to detect glycine originating from neuronal, phasic release as compared to, for example, capillary electrophoresis coupled with laser‐induced fluorescence detection (15 s), a method previously employed to demonstrate accumbal glycine elevation in anticipation to reward (ethanol‐gel or plain gel) in rats (Li et al. 2008). Further, parallel GlyT1‐activity, which removes glycine from the extracellular space, may have concealed tentative effects following GlyT2‐blockade as GlyT1 is reported to be more abundant than GlyT2 in the nAc (Zeilhofer et al. 2005). The latter hypothesis is supported by a previous study in which combined, systemic GlyT1‐ and GlyT2‐blockade in threshold doses produced additive effects on cerebrospinal levels of glycine and analgesia in rats (Mohammadzadeh et al. 2021). Nonetheless, combined GlyT1‐ and GlyT2‐blockade in the present study did not produce additive effects on accumbal glycine, nor DA output, thus contradicting this theory. Notably, the magnitude in DA elevation appears to be more pronounced following GlyT1‐ (sustained elevation by 20%–33% in the present study) as compared to GlyT2‐inhibition (transient peak of approx. 20%), inferring that extrasynaptic rather than synaptic GlyRs are involved in regulating basal DA levels.

Despite the modest effects on basal accumbal DA levels, rats treated with NAGly presented a lower relative alcohol intake (after versus before the alcohol deprivation) as compared to vehicle‐treated controls and completely abolished the ADE, in line with previous studies on GlyT1‐inhibitors (Olsson et al. 2023; Vengeliene et al. 2018). Water intake was, in contrast, slightly decreased following the alcohol deprivation period in the vehicle‐treated group but not in the NAGly‐treated group, possibly reflecting a reciprocal change in fluid consumption associated with the concomitant increase in alcohol intake. In light of our findings demonstrating a relatively high concentration of GlyT2 in the lateral septum (LS), a limbic brain region also implicated in alcohol‐related behavior and reward (Olds and Milner 1954; Ryabinin et al. 2008), it is plausible that effects on alcohol consummatory behavior observed here may derive from targeting this region too. Indeed, previous studies have demonstrated that the LS modulates basal (Sheehan et al. 2004) and alcohol‐induced (Jonsson et al. 2017) DA output in the nAc and the presence of GlyR‐expressing, GABA‐ergic neurons projecting from the LS to the nAc and VTA (Jonsson et al. 2017). Another plausible mechanism of action for NAGly in enhancing DA output and reducing alcohol intake derives from its dual interference with glycinergic transmission. NAGly, an endogenous lipid hypothesized to act as an endogenous analgesic in the spinal cord, enhances glycinergic neurotransmission by both partial GlyT2‐inhibition and by acting as a positive allosteric modulator for α1‐ and α1β‐GlyRs while found to be inactive or modestly inhibitory at α2‐ and α3‐ GlyR constellations (Gallagher et al. 2022). An explanatory model that involves enhanced glycinergic neurotransmission by allosteric modulation of GlyRs would also be congruent with observations made in the present study, that is, (I) that extracellular accumbal DA levels were raised while glycine levels remained unaltered following NAGly treatment and (2) that NAGly, but not the full GlyT2‐inhibitor Org 25,543, to the best of our knowledge devoid of modulatory actions directly onto GlyRs, slightly raised DA levels in the nAc. Accordingly, NAGly may also be employed for subunit‐specific modulation of glycinergic transmission, although both α1‐ (Araya et al. 2023; Muñoz et al. 2020) and α2‐ (Gallegos et al. 2021) GlyRs appear to be involved in regulating alcohol intake in mice.

Noteworthy, findings from the present study also suggest that reversible rather than irreversible GlyT1‐inhibitors, or glycine itself, are more reliable in enhancing mesoaccumbal DA activity. Indeed, the reversible GlyT1‐inhibitor bitopertin produced an accumbal DA elevation by 20%–33%, sustained for 180 min in the full group of animals, as compared to previous studies for the irreversible GlyT1‐inhibitor Org 25,935 (Lidö et al. 2009) or glycine itself (Olsson et al. 2021), in which alterations in DA levels were only observed in a subpopulation of animals and/or were less pronounced. These differences may stem from that irreversible GlyT1‐inhibitors and exogenously applied glycine produce a more prolonged and presumably excessive elevation in glycine levels as compared to reversible GlyT1‐inhibitors, whereby GlyRs are more prone to desensitization that overall reduces their activity. Moreover, a reversible GlyT1‐inhibitor structurally similar to bitopertin has also been shown to reduce relapse‐like drinking and cue‐induced reinstatement in rats (Vengeliene et al. 2018) thus suggesting this drug class, which is devoid of adverse side effects in rats (Alberati et al. 2012) or humans (Hofmann et al. 2016), to be preferable within glycinergic compounds, for alleviating compromised mesoaccumbal DA activity and ultimately reduce alcohol intake.

In similarity with reversible GlyT1‐inhibitors being preferable over their irreversible counterpart, the present study also supports the notion that partial rather than full GlyT2‐inhibitors are preferred in order to enhance GlyR‐induced DA release in the nAc. Indeed, only the partial GlyT2‐inhibitors slightly raised accumbal DA levels, while the full GlyT2‐inhibitor at the higher dose produced an apparent slight reduction in DA levels, as indicated by a significant time × treatment interaction. This discrepancy may be related to how full and partial GlyT2 inhibition affect glycinergic neurotransmission, which in turn is hypothesized to modulate accumbal DA levels. Indeed, high doses of full GlyT2‐inhibitors have previously been shown to produce an initial increase in glycinergic neurotransmission followed by a subsequent reduction, presumably related to depletion of glycine content in vesicles at the presynaptic terminal (Cioffi 2021).

Important limitations to the study should also be discussed. In contrast to previous studies on GlyT1‐inhibitors and exogenously delivered glycine, the present study did not invoke local perfusion of GlyT2‐inhibitors into the nAc, reversal of effects using the GlyR‐antagonist strychnine, nor microdialysis experiments and evaluation of alcohol consummatory behavior within the same animal. Accordingly, a causal relationship between raised accumbal DA output and reduced alcohol intake in rats, and that these events derive from interference with accumbal GlyT2s, cannot be confirmed. However, a mechanistic framework that links GlyR‐mediated alterations in DA levels within the nAc to reduced voluntary alcohol consumption has indeed been demonstrated for treatment with glycine and GlyT1‐inhibitors (Söderpalm et al. 2017). In conjunction with previous (Muñoz et al. 2018) and present IHC data showing that GlyT2s are present in the nAc, and in light of the fact that the GlyT2‐inhibitors employed here previously have been shown to produce analgesic effects (Burstein 1999; Pang et al. 2012), that is, to reach targets in the CNS following systemic administration, we therefore hypothesize that the present observations are, at least partly, driven by enhanced GlyR‐activity in the nAc, in turn mediated by partial inhibition of accumbal GlyT2s. Finally, the present study only included male rats, thereby neglecting tentative sex‐related differences in neurochemical responses and alcohol consummatory behavior and limiting its translational validity.

To conclude, new pharmacological targets for the treatment of AUD are warranted and accumbal GlyRs have emerged as such, given its involvement in neurobiological correlates for negative and positive reinforcement to consume alcohol, that is, altered basal and alcohol‐induced DA output. This study confirms the presence of GlyT2 in the nAc, presumably located on glycinergic axons, and suggests that GlyT2s and synaptic GlyRs are involved in regulating basal accumbal DA levels, but to a lesser extent compared to GlyT1s and extrasynaptic GlyRs. Moreover, GlyT2s appear to be involved in regulating voluntary alcohol intake, probably mediated by GlyRs within or outside of the nAc, and ought to be explored in future studies that invoke, for example, reversed microdialysis into the nAc and the LS and mapping of glycinergic projections using, for example, retrograde tracing techniques. In line with previous work on glycinergic transmission in animal models of pain, studies that explore tentative synergistic effects on accumbal DA output and alcohol intake following combined GlyT1‐ and GlyT2‐blockade in threshold doses, that is, lower doses than applied here, are also warranted. Overall, findings from the present study suggest that partial GlyT2‐inhibition, alongside GlyT1‐inhibition and application of exogenous glycine, may be utilized to enhance GlyR‐mediated accumbal DA output, reduce alcohol intake and ultimately represent a new treatment principle for AUD.

## Funding

This work was supported by Göteborgs Läkaresällskap, GLS‐999159. Stiftelsen Professor Bror Gadelius Minnesfond. Stiftelserna Wilhelm och Martina Lundgrens, 2024‐SA‐4436. Grants from the Swedish State under the agreement between the Swedish Government and county council Region Västra Götaland, the ALF agreement, ALFGBG‐984234. Stiftelsen Lars Hiertas Minne.

## Conflicts of Interest

The authors declare no conflicts of interest.

## Data Availability

The data that support the findings of this study are available from the corresponding author upon reasonable request.
